# Supplementary figures and images for: De novo synthesized polyunsaturated fatty acids operate as both host immunomodulators and nutrients for Mycobacterium tuberculosis
Source: eLife. 2021 Dec 24;10:e71946. doi: 10.7554/eLife.71946 (PMC8752091; doi:10.7554/eLife.71946)

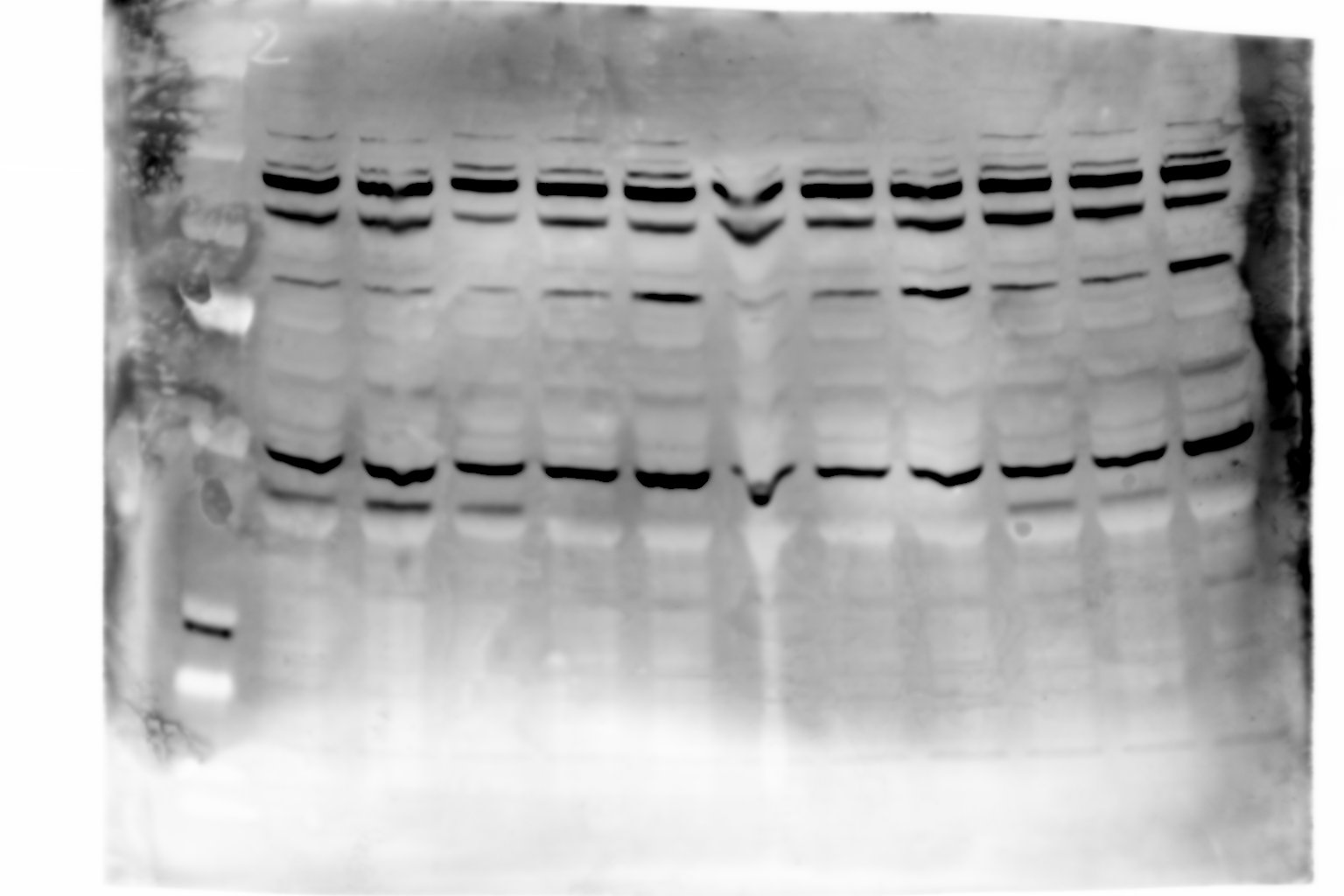

Supplement: Figure 4—figure supplement 1—source data 1. [file elife-71946-fig4-figsupp1-data1.jpg]

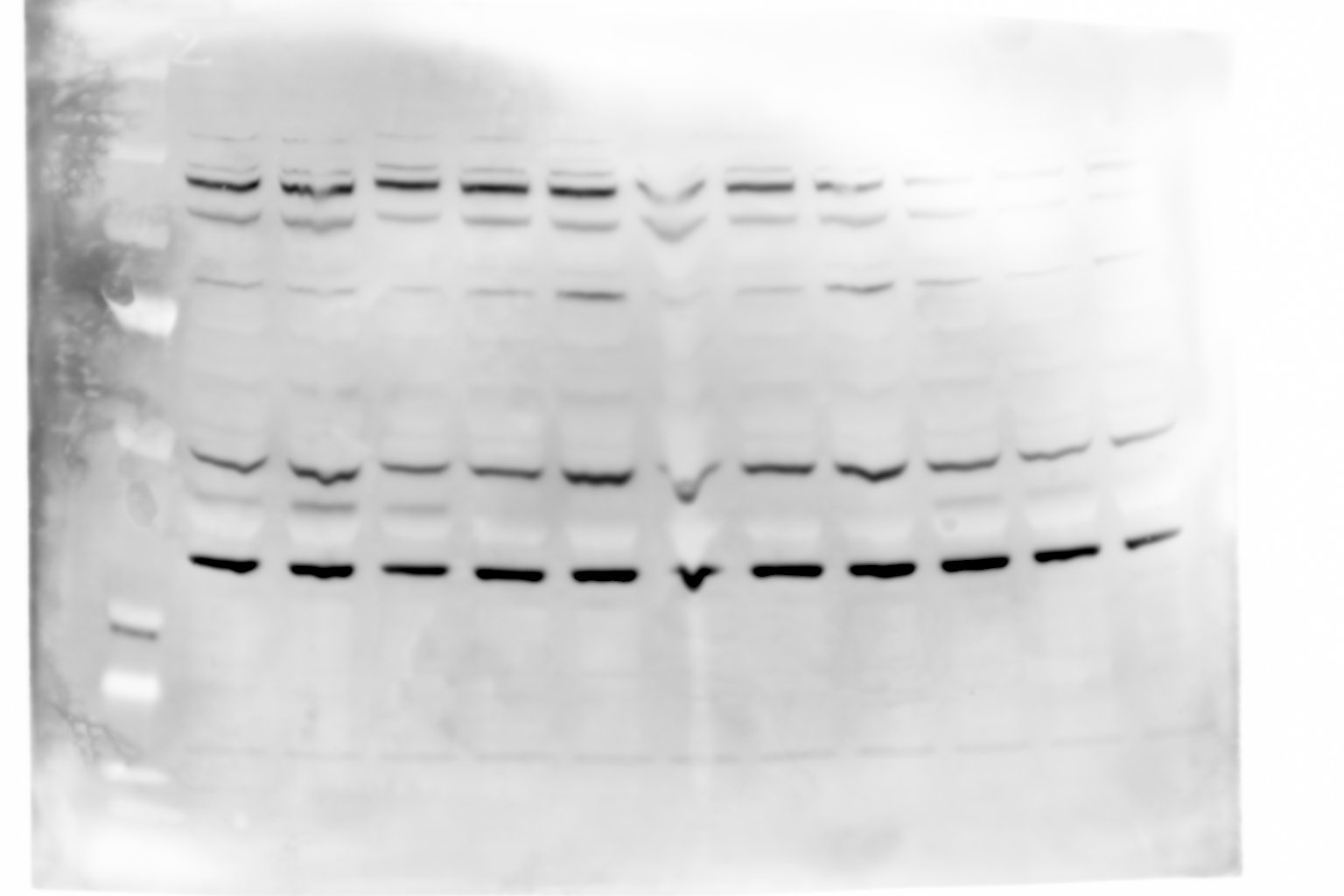

Supplement: Figure 4—figure supplement 1—source data 2. [file elife-71946-fig4-figsupp1-data2.jpg]

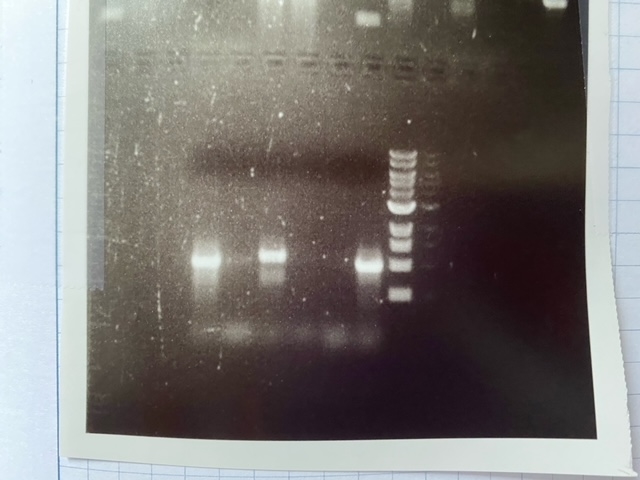

Supplement: Figure 5—figure supplement 1—source data 1. [file elife-71946-fig5-figsupp1-data1.jpg]

primers    1+kmR    4+kmF    1+7  
                 1    2    3    4    5    6    Ladder

1, 3, 5: Mutant  
2, 4, 6: WT

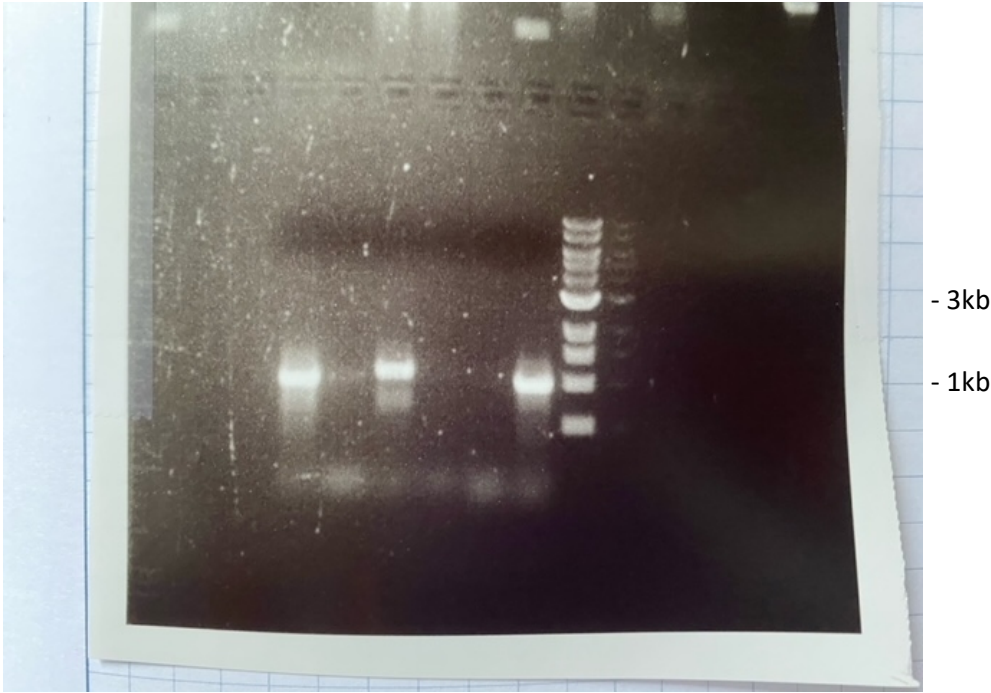

Supplement: Figure 5—figure supplement 1—source data 2. [file elife-71946-fig5-figsupp1-data2.pdf]
