# Supplementary material for: De novo synthesized polyunsaturated fatty acids operate as both host immunomodulators and nutrients for Mycobacterium tuberculosis
Source: eLife. 2021 Dec 24;10:e71946. doi: 10.7554/eLife.71946 (PMC8752091; doi:10.7554/eLife.71946)
Supplement: Figure 4—figure supplement 1—source data 3. [file elife-71946-fig4-figsupp1-data3.pdf]

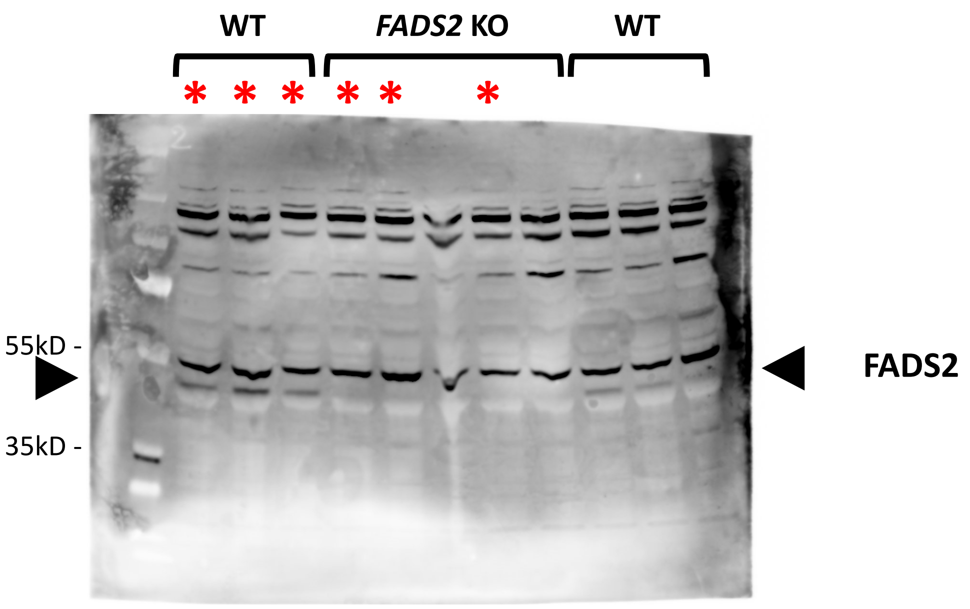

The same blot was stripped after FADS2 detection, and incubated with anti-GAPDH antibody followed by the secondary antibody:

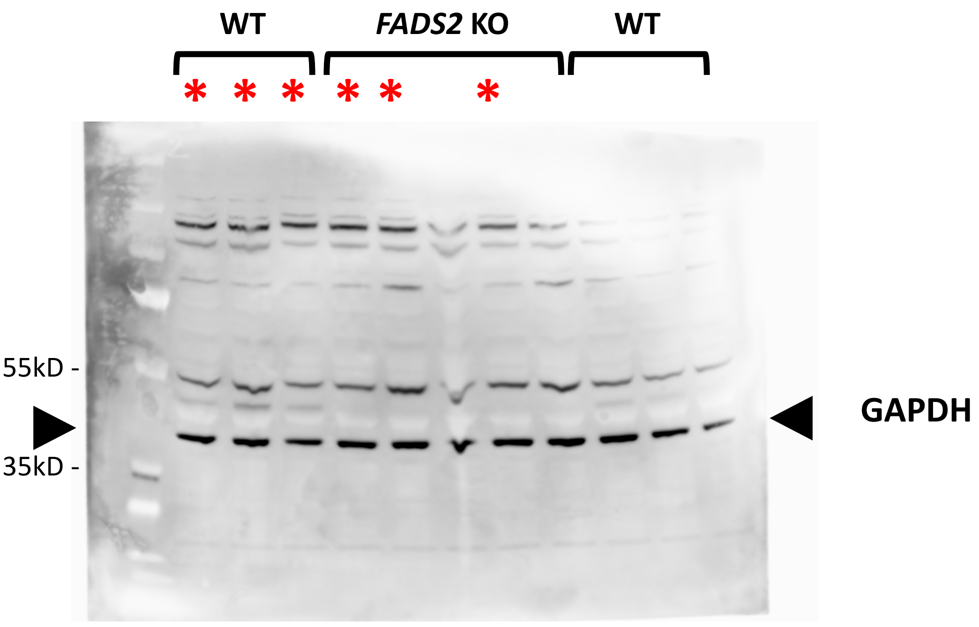

* : independent clones selected for the study
