# Supplementary material for: De novo synthesized polyunsaturated fatty acids operate as both host immunomodulators and nutrients for Mycobacterium tuberculosis
Source: eLife. 2021 Dec 24;10:e71946. doi: 10.7554/eLife.71946 (PMC8752091; doi:10.7554/eLife.71946)
Supplement: Supplementary file 1. [file elife-71946-supp1.docx]

| ***FA metabolism*** | ***Chimio/Cytokines*** | ***Antibacterial*** |
| --- | --- | --- |
| *Acot1* | *Ccl2* | *Gbp1* |
| *Acot2* | *Ccl3* | *Gbp6* |
| *Acox1* | *Ccl5* | *Gbp7* |
| *Acox3* | *Cxcl10* | *Irg1* |
| *Cd36* | *Cxcl3* | *Irgm1* |
| *Cpt1a* | *Ifnb* | *Irgm2* |
| *Elovl5* | *Ifng* | ***ROS/ROI/RNS*** |
| *Elovl6* | *Il10* | *Cybb* |
| *Fads1* | *Il12a* | *Gpx3* |
| *Fads2* | *Il12b* | *Gpx4* |
| *Fasn* | *Il18* | *Ncf4* |
| *Lxra (Nr1h3)* | *Il1b* | *Nos2* |
| *Lxrb (Nr1h2)* | *Il6* | *Nox1* |
| *Ppara* | *Il8* | *Sod2* |
| *Pparg* | *Tfn* | ***Receptors*** |
| *Scd1* | *Tgfb* | *Cd54* |
| *Scd2* | ***Signaling*** | *Cd64* |
| *Srebf1* | *Aim2* | *Cd86* |
| ***Lipid inflammatory mediators*** | *Casp1* | *Clec4e* |
| *Ptger2* | *Cebpb* | *Ifngr1* |
| *Alox5* | *Ciita* | *Ifngr1* |
| *Lta* | *Hif1a* | *Ifngr2* |
| *Ltb* | *Irak3* | *Ifngr2* |
| *Ltb4r1* | *Irf1* | *Marco* |
| *Ltb4r2* | *Irf3* | *Mrc1* |
| *Ltbr* | *Irf7* | *Nod1* |
| *Ptger4* | *Jak1* | *Nod2* |
| *Ptges* | *Mapk1* | *Tlr2* |
| *Ptgs2* | *Mapk14* | *Tlr4* |
| ***Cholesterol metabolism*** | *Myd88* | *Trem1* |
| *Ch25h* | *Nfkb1* | ***Iron metabolism*** |
| *Cyp27a1* | *Nfkb2* | *Scl40a1* |
| *Srebf2* | *Nlrp3* | *Slc11a1* |
| ***Lipid droplets*** | *Socs1* | *Slc11a2* |
| *Plin2* | *Socs3* | *Tfrc* |
| *Pnpla2* | *Tbk1* | ***Housekeeping*** |
| ***Antimicrobial peptides & Vitamin D*** | *Traf6* | *G6pdx* |
| *Camp* | *Trif* | *Hprt* |
| *Cyp27b1* | ***Autophagy*** | *Oaz1* |
| *Defb1* | *Atg5* | *Ppia* |
| *Vdr* | *Lipa* | *Rpl19* |
| ***Phagosome acidification*** | *Map1Lc3b* |  |
| *Cish* |  |  |
