# Supplementary material for: De novo synthesized polyunsaturated fatty acids operate as both host immunomodulators and nutrients for Mycobacterium tuberculosis
Source: eLife. 2021 Dec 24;10:e71946. doi: 10.7554/eLife.71946 (PMC8752091; doi:10.7554/eLife.71946)
Supplement: Supplementary file 2. — DraIII and Van91I restriction sites introduced into the primers are indicated in red and green, respectively. [file elife-71946-supp2.docx]

| A1 | CGATCAGGTTGGGCACTG | *AES LucA (Rv3723)* |
| --- | --- | --- |
| A2 | TTTCACTTCGTGGGCATCCAGGCTGAGCC | *AES LucA (Rv3723)* |
| A3 | TTTCACAGAGTGCGCAGGAGGACGAGGAG | *AES LucA (Rv3723)* |
| A4 | CGCAGTTCTGGCTACAGG | *AES LucA (Rv3723)* |
| A5 | GCAGTTCGGCGAAGATAGC | *AES LucA (Rv3723)* |
| A6 | CGGCACACAACTGAAGCG | *AES LucA (Rv3723)* |
| B1 | CGAACCCAGACGGTCCAC | *AES mce1D* |
| B2 | TTTCACTTCGTGCGAGCAAGCTGTCGAATTG | *AES mce1D* |
| B3 | TTTCACAGAGTGGGGTACAAGGACACCACGG | *AES mce1D* |
| B4 | CGTTGCTGGTTGAGCTCG | *AES mce1D* |
| B5 | CCGATTCCACAGGAGGCTC | *AES mce1D* |
| B6 | GGTGAAGGTGTCGAGCTTG | *AES mce1D* |
| C1 | CTACTCACGGATGTTTGATCG | *AES mceG (mk1)* |
| C2 | TTTCCATTTCTTGGCACGCTTACGCATCCC | *AES mceG (mk1)* |
| C3 | TTTCACAGAGTGGCAAGCATTTGGTGATGTTCG | *AES mceG (mk1)* |
| C4 | GGACGAGTTCGACCAGATG | *AES mceG (mk1)* |
| C5 | CTCGCTGGATCGCTGGAC | *AES mceG (mk1)* |
| C6 | GGATGTTTGGTGACGTCTAC | *AES mceG (mk1)* |
| D1 | GGAGCAGAGCCAGCCAC | *AES Rv0200* |
| D2 | TTTCACTTCGTGGTGGCAAGATGCACCACTTC | *AES Rv0200* |
| D3 | TTTCACAGAGTGGGGTGACCTTGACCAAGCG | *AES Rv0200* |
| D4 | GCTTCAGACAACCACCTGG | *AES Rv0200* |
| D5 | AAGAAGATGCGGAAGACGAC | *AES Rv0200* |
| D6 | GGGGTGCAGACTCACCG | *AES Rv0200* |
| F1 | CGCAAGGGTGGTCGATCAG | *AES yrbE1A* |
| F2 | TTTCACTTCGTGGCGTGAAGATCAACAGCACC | *AES yrbE1A* |
| F3 | TTTCACAGAGTGGGCATCGATCCCATCCACC | *AES yrbE1A* |
| F4 | GACGCAGGAACGTTTGAAAG | *AES yrbE1A* |
| F5 | CAGAACATGAACGATGTCCC | *AES yrbE1A* |
| F6 | GCTCGTAGGTGCCGTTCG | *AES yrbE1A* |
| G1 | GGTGTCTGGTGCCGTTTCG | *AES yrbE2A* |
| G2 | TTTCCATTTCTTGGCAGCAACGAGACGCTGG | *AES yrbE2A* |
| G3 | TTTCACAGAGTGGGAAGAGATCGATGCGATGG | *AES yrbE2A* |
| G4 | GTTGTGACCACCTGACCAG | *AES yrbE2A* |
| G5 | GGACAACTTAGCCCGATAAC | *AES yrbE2A* |
| G6 | CAGAGGTGAAAGCCATGTCG | *AES yrbE2A* |
| H1 | GTCGTTCATGGTGACGTTTC | *AES yrbE3A* |
| H2 | TTTCACTTCGTGCATGGGATCGTCATCAACAC | *AES yrbE3A* |
| H3 | TTTCACAGAGTGGACGTCGTCATTTCGATGATC | *AES yrbE3A* |
| H4 | CGTCTCATGTCACTAGGTCG | *AES yrbE3A* |
| H5 | GAGGCTGTGCTGCTGCCAAG | *AES yrbE3A* |
| H6 | CAGGTGAAAGTTGGGCGAC | *AES yrbE3A* |
| I1 | GGATGCTGTTCAACATGTCC | *AES yrbE4A* |
| I2 | TTGTTCATGACGTCGGGACTAGCGAAAC | *AES yrbE4A* |
| I3 | TAATTCATGACAACGGTCTGACCGTGCTC | *AES yrbE4A* |
| I4 | GATGACAACAACCACGATCAG | *AES yrbE4A* |
| I5 | CCGACGAGGAGTGGGACG | *AES yrbE4A* |
| I6 | GATCAGCGAGGTCCGTAC | *AES yrbE4A* |
| ki1 | AGTCCCGACGTCATGAACAATAAAACTGTCTGC | *AES yrbE4A* |
| ki2 | CAGACCGTTGTCATGAATTAATTCTTAGAAAAACTC | *AES yrbE4A* |
| k1 | TTTCACGAAGTGTCATGAACAATAAAACTGTCTGC | *DraIII kmR* |
| k2 | TTTCACTCTGTGTCATGAATTAATTCTTAGAAAAACT | *DraIII kmR* |
| Z8 | ATATCTAGAATGATGCTAGGACAATTGGAAAG | *Complementation mce1 locus* |
| Z9 | ATAGATATCGGTCAGTCCTGCTCCTCG | *Complementation mce1 locus* |
| M1 | TTACTCACAGGGAGTAACGGAGACCACAGGAATCTACCCGATGGCCAGCCAGGAGTGTAA  GGCATCCGCACATTTAACCCTTCGTCAATAGCGGGCAAACGGGAACGGCAATGCTCCTGG | *Complementation mce1 locus* |
| M2 | TGACGCGCGGTGGCTGGTGACCAGGATGACGACCATCACCTCGCTGGATTTGACGCCGCA  GGTGTAGCGGGACCGAGCCCGCCGGCGCTGCGAAGCCGATGCTAGGACAATTGGAAAGC | *Complementation mce1 locus* |
| KmF | GCCATCCTATGGAACTGCC | *screening mutant* |
| KmR | GCCTAGAGCAAGACGTTTCC | *screening mutant* |
| A7 | CCGTAGATCCCGAACAACC | *screening mutant* |
| B7 | GGTTCTTGGCGAAGAACGG | *screening mutant* |
| C7 | GGGTCAAGGACCAGGG | *screening mutant* |
| D7 | GGAGAATGACGCGACCG | *screening mutant* |
| F7 | GGAACCGGAGATGTCTGCC | *screening mutant* |
| G7 | GGTCAACGGAATCGACACC | *screening mutant* |
| H7 | GCAAGACGTTGAAGAGAAACC | *screening mutant* |
| I7 | CAGGACCGTGAACGGAATG | *screening mutant* |
| 78 | CCCTACCTCTCCGAGCG | *screening mutant* |
| Z1 | GAGACCTGGGCACCAGC | *screening mutant* |
| Z3 | GAGACACAACGTGGCTTTCC | *screening mutant* |
