# Supplementary material for: De novo synthesized polyunsaturated fatty acids operate as both host immunomodulators and nutrients for Mycobacterium tuberculosis
Source: eLife. 2021 Dec 24;10:e71946. doi: 10.7554/eLife.71946 (PMC8752091; doi:10.7554/eLife.71946)
Supplement: Supplementary file 3. [file elife-71946-supp3.docx]

| **Gene** |  | **Sequence (5’ -> 3’)** | **Reference** |
| --- | --- | --- | --- |
| *Fasn* | Fwd | CCCTTGATGAAGAGGGATCA | NM_007988 |
|  | Rev | GAACAAGGCGTTAGGGTTGA |  |
| *Scd2* | Fwd | CCCCCTCCGCTTGGCTAGGG | NM_009128 |
|  | Rev | AGCTTGCAGCCACCGGTGTC |  |
| *Fads1* | Fwd | TGGTGCCCTTCATCCTCTGT | NM_146094 |
|  | Rev | GGTGCCCAAAGTCATGCTGTA |  |
| *Fads2* | Fwd | TCCTGTCCCACATCATCGTCATGG | NM_019699 |
|  | Rev | GCTTGGGCCTGAGAGGTAGCGA |  |
| *Elovl5* | Fwd | ATGGAACATTTCGATGCGTCA | NM_134255 |
|  | Rev | GTCCCAGCCATACAATGAGTAAG |  |
| *Lxra* | Fwd | CCTTCCTCAAGGACTTCAGTTACAA | NM_013839 |
|  | Rev | CATGGCTCTGGAGAACTCAAAGAT |  |
| *Pparg* | Fwd | AGAGTCTGCTGATCTGCGAG | NM_011146 |
|  | Rev | GGCATACTCTGTGATCTCTTG |  |
| *Srebf1* | Fwd | AGGCCATCGACTACATCCG | NM_011480 |
|  | Rev | ATCCATAGACACATCTGTGCCTC |  |
| *Dhcr24* | Fwd | CATCGTCCCACAAGTATG | NM_053272 |
|  | Rev | CTCTACGTCGTCCGTCA |  |
| *Ch25h* | Fwd | TGCTACAACGGTTCGGAGC | NM_009890 |
|  | Rev | AGAAGCCCACGTAAGTGATGAT |  |
| *Il1b* | Fwd | GCAACTGTTCCTGAACTCAACT | NM_008361 |
|  | Rev | ATCTTTTGGGGTCCGTCAACT |  |
| *Il6* | Fwd | AGTTGCCTTCTTGGGACTGA | NM_031168 |
|  | Rev | TCCACGATTTCCCAGAGAAC |  |
| *Tnf* | Fwd | CTGGGACAGTGACCTGGACT | NM_013693 |
|  | Rev | GCACCTCAGGGAAGAGTCTG |  |
| *Ptgs2* | Fwd | TTCAACACACTCTATCACTGGC | NM_011198 |
|  | Rev | AGAAGCGTTTGCGGTACTCAT |  |
| *Nos2* | Fwd | ACATCGACCCGTCCACAGTAT | NM_010927 |
|  | Rev | CAGAGGGGTAGGCTTGTCTC |  |
| *Syt7* | Fwd | CCGTCAGCCTTAGCGTCAC | NM_018801 |
|  | Rev | GCAGGCAACTTGATGGCTTTC |  |
| *Rpl19* | Fwd | TACTGCCAATGCTCGG | NM_009078 |
|  | Rev | AACACATTCCCTTTGACC |  |
